# *Hypericum perforatum* Oil and Vitamin A Palmitate-Loaded Gelatin Nanofibers Cross-Linked by Tannic Acid as Wound Dressings

**DOI:** 10.1021/acsomega.3c02967

**Published:** 2023-06-26

**Authors:** Aysen Akturk, Funda Nur Kasikci, Dilara Nur Dikmetas, Funda Karbancioglu-Guler, Melek Erol-Taygun

**Affiliations:** † Department of Chemical Engineering, Istanbul Technical University, Maslak, Istanbul 34449, Turkey; ‡ Department of Food Engineering, Istanbul Technical University, Maslak, Istanbul 34449, Turkey

## Abstract

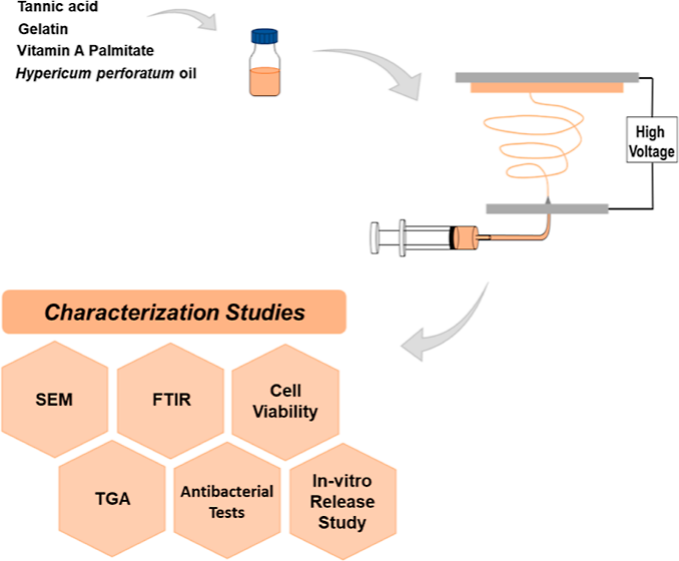

Recent studies in wound dressing applications offer new therapies to promote the wound healing process. The main strategy of this study is to combine the traditional perspective of using medicinal oils with polymeric scaffolds manufactured by an engineering approach to fabricate a potential tissue engineering product that provides both new tissue formation and wound healing. Thus, *Hypericum perforatum* oil (HPO) and vitamin A palmitate (VAP) incorporated gelatin (Gt) nanofibrous scaffolds were successfully prepared by the electrospinning method. Tannic acid (TA) was used as the cross-linking agent. The amounts of VAP and HPO loaded in the base Gt solution [15% w/v in 4:6 v/v acetic acid/deionized water] were 5 and 50 wt % (based on the weight of Gt), respectively. The obtained scaffolds were studied regarding their microstructure, chemical structure, thermal stability, antibacterial activity, in vitro release study, and cellular proliferation assay. In the light of these studies, it was determined that VAP and HPO were incorporated successfully in Gt nanofibers cross-linked with TA. Release kinetic tests confirmed that the patterns of TA and VAP release were consistent with the Higuchi model, whereas HPO release was consistent with the first-order kinetic model. In addition, this membrane was biocompatible with L929 fibroblast cells and had antibacterial activity and thermal stability. This preliminary study suggests potential applicability of the proposed dressing to treat skin wounds in clinics.

## 1 Introduction

Wound treatment is a significant public health issue and an economic burden. The wound care standard includes removing dead tissue, cleansing and disinfecting the wound site, and dressing it to prevent infection and speed up the healing process.^1^ Electrospun nanofiber-based wound dressings have emerged as one of the most promising therapeutic options for wound healing management, as they exhibit structural properties quite similar to those offered by natural skin’s extracellular matrix (ECM).^2^ The electrospun nanofiber mats may readily integrate antimicrobial (e.g., antibiotics, silver-based compounds, and natural extracts) and biological molecules (e.g., growth factors, vitamins and anti-inflammatory medications, and antioxidants) to promote a more rapid and robust wound healing process for multifunctional dressings for local delivery.^2−5^

Gelatin (Gt) is a protein-based biopolymer derived from partial hydrolysis of natural collagens, which are structural proteins in the animals’ skin, tendons, cartilage, and bone.^6^ It is widely utilized in the food, printing, adhesives, and pharmaceutical industries, as well as in drug delivery systems, sealants for vascular grafts and patches, wound healing, tissue engineering, and artificial skin engineering due to its several advantages, including biological origin, biodegradability, biocompatibility, and commercial availability at a comparatively low cost.^7,8^ Additionally, it may be used to encapsulate bioactive compounds such as essential oils (basil essential oil, bergamot, lemongrass essential oil, *Origanum vulgare* L. essential oil, and orange leaf essential oil) and vitamins (vitamin C, vitamin B12, and vitamin D3).^9−12^

Despite its promise, Gt, on the other hand, is water-soluble, has low mechanical strength, and has poor thermal stability in processing, which restricts its applicability.^6,7^ Generally, cross-linking is capable of altering the reticular structure of Gt, improving its functional characteristics and expanding its industrial uses.^6^ In this context, numerous substances, including aldehydes (glutaraldehyde and formaldehyde), natural cross-linking agents (genipin and dextran), and some enzymes (transglutaminase), as well as phenolic compounds such as catechin, caffeic acid, gallic acid, and tannic acid (TA), have been used to cross-link and enhance the activity of Gt.^6,12^ Phenolics are natural compounds that are abundant in a broad range of plants. They are non-toxic and usually considered safe and the antioxidant. TA possesses a significant quantity of hydroxyl groups in comparison to other phenolic compounds to strongly bind Gt. It has a bacteriostatic action against food-borne infections and is also a powerful antioxidant.^7^ The use of Gt and tannins as raw materials in the fabrication of electrospun nanofibers may be a viable alternative to hazardous aldehydes and costly natural cross-linking agents and enzymes.^6^

Vitamin A is a fat-soluble crucial vitamin for human health and consists of retinol, retinal, retinoic acid, and several provitamin A carotenoids.^13,14^ As has been reported previously, the most prominent property of vitamin A is its antioxidant capacity, and therefore it may show potential for treatments of cancer, diabetes, dermatological disorders, and cardiovascular diseases.^15^ It also takes a significant role in the treatment of skin recovery, retinitis, etc.^14^ It can increase collagen deposition in the repair zone of a wound. However, it suffers from stability issues. Thus, its derivatives have been developed: vitamin A palmitate (VAP) is a lipid-soluble substance used for the treatment of skin diseases and offers greater stability than vitamin A. It can be converted into vitamin A (retinol) in physiological conditions.^4^ Therefore, recent studies target incorporating VAP to different nanofibrous wound dressings.^4,13^

*Hypericum perforatum*, which belongs to the *Hypericaceae* family, is one of the most consumed valuable medicinal plants in the world.^16,17^ It has been used in a variety of topical and oral applications such as cuts, burns, depression, hemorrhoids, diabetes, and gastrointestinal ulcers due to its constituents such as hypericins, hyperforins, and flavonoids.^5,16−20^*H. perforatum* oil (HPO, also known as centaury oil or St. John’s Wort oil) is commonly used in alternative medicine for wound healing.^5^ For this reason, several researchers have used HPO in nanofiber wound dressings as a way to improve their healing properties. Studies indicated that HPO-loaded membranes have good biocompatibility and antibacterial and mechanical properties for usage in biomedical applications.^5,16−20^

In this study, an *H. perforatum* oil and VAP incorporated Gt nanocomposite material was designed as a wound dressing material for the first time. TA was used as an environmentally friendly crosslinking agent that provides structural integrity to the obtained wound dressing. The morphology, thermal and physical characteristics, chemical composition, and cellular proliferation of the electrospun wound dressings were studied. The obtained electrospun nanocomposite material demonstrated antibacterial activity and has the potential for use in a variety of biomedical applications, particularly wound dressing, due to the combination of the antibacterial activity, healing effect, and thermal stability of the TA, HPO, and VAP in the Gt nanofibrous structure.

## 2 Materials and Methods

### 2.1 Materials

Gt (type A, from porcine skin, gel strength 300, G2500), TA (ACS reagent), and VAP (potency: ≥1,700,000 USP units per g) were purchased from Sigma-Aldrich and used as received. Acetic acid (glacial) and Tween 80 [CAS 9005-65-6, pH 5–7 (50 g/L, H_2_O, 20 °C)] were obtained from Merck, and organic maceration oil St. John’s Wort [*Olea Europaea* (Olive) fruit oil, *H. perforatum* flower extract] was purchased from Florame. All solutions were prepared using deionized water.

### 2.2 Preparation of Gt/HPO and Gt/HPO/VAP Mixtures

Four polymer solutions, namely, Gt, Gt–TA, Gt–TA–HPO, and Gt–TA–HPO–VAP were prepared. For each solution, acetic acid and deionized water were in a volumetric ratio of 4/6 (v/v). Gt solution (15% w/v) was prepared in acetic acid and deionized water at 30 °C for 1 h. TA was used as the crosslinking agent. TA (15% w/w, with respect to the polymer) was added to the Gt solution to prepare a Gt–TA solution. Tween 80 was added to this polymer solution at a concentration of 0.1% (w/w, with respect to the polymer) for the preparation of HPO oil-loaded Gt nanofibers. HPO with a weight ratio of 50% (w/w, with respect to the polymer) was added to the Gt–TA polymer solution to obtain a Gt–TA–HPO mixture. The Gt–TA–HPO–VAP mixture was prepared by mixing VAP at a concentration of 5% (w/w, with respect to the polymer).

### 2.3 Electrospinning

**Figure 1:**
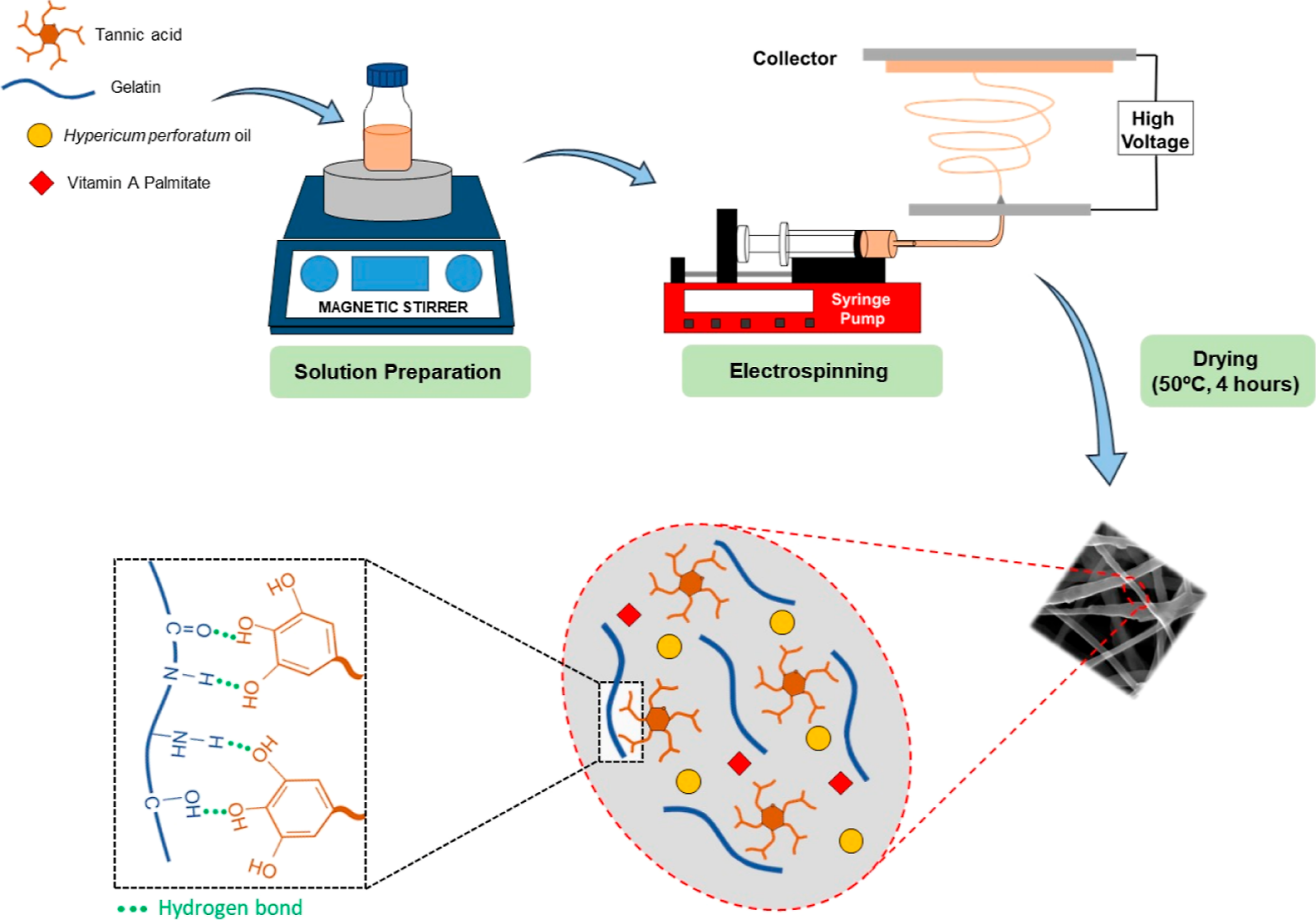
Schematic illustration of the preparation of HPO and VAP loaded Gt nanofiber membranes and Gt–TA crosslinking interactions.

The Gt, Gt–TA, Gt–TA–HPO, and Gt–TA–HPO–VAP mixtures were loaded into syringes fixed to a syringe pump and fed from the syringe to a needle perpendicular to a metal plate collector. The nanofibers were deposited on the collector plate wrapped with aluminum foil. The operating parameters were a needle-to-collector distance of 16 cm, an applied voltage of 20 kV, and a flow rate of 1.0 mL/h. The entire procedure was carried out using an electrospinning device (Nanospinner 24 Touch, Inovenso) under ambient conditions. After the electrospinning process, the fibers were dried at 50 °C for 4 h. As hydrogen bonds become more dynamic at high temperature, this heat treatment was used to enhance the crosslinking process with TA.^21^ The nanofiber fabrication procedure and Gt–TA crosslinking interactions are given in Figure 1.

### 2.4 Characterization of Nanofibers

The electrospun nanofibers were platinum sputtered for 120 s (SC7620 sputter coater, Quorum Technologies Ltd, United Kingdom) and analyzed with scanning electron microscopy (SEM, Quanta FEG 250), and ImageJ software was used to measure the fiber diameter. The molecular structures of the nanofiber membranes were studied with the aid of Fourier transform infrared (FTIR, Jasco FT/IR 4700) at 4000–500 cm^–1^. In order to determine the thermal properties of these nanofiber membranes, thermogravimetric (TGA) and derivative thermogravimetric (DTG) analyses were performed with a Hitachi STA200 (TG-DTA/DSC) device between 30 and 600 °C at a heating rate of 10 °C/min under a nitrogen atmosphere.

### 2.5 Antibacterial Activity

Antimicrobial tests were performed on a Gram-negative (*Escherichia coli* ATTC 25922) and a Gram-positive (*Staphylococcus aureus* ATTC 25923) bacteria using the disc diffusion method and optical density (OD) measurement.

Bacteria were grown in Mueller Hinton broth (MHB) (Merck: 110293) by incubation at 37 °C for 24 h. At the end of the incubation, the concentrations of bacteria were adjusted to approximately 10^5^ cfu/mL for disc diffusion analysis. Before the analysis, nanofibers were cut into 6 mm diameter discs. Both sides of the discs were kept separately at 266 nm in a UV cabinet for 20 min. 100 μL of the bacterial cultures were spread on Mueller Hinton agar (MHA) (Merck: 103872) plates. After that, 6 mm nanofiber samples were placed on the media under aseptic conditions, and the Petri plates were incubated at 37 °C for 24 h. At the end of the incubation, antibacterial properties of the samples were evaluated by the measurement of inhibition zones. Antimicrobial tests were performed in three parallel studies. Standard bioanalysis antimicrobial susceptibility testing discs ampicillin (10 μg), gentamycin (10 μg), kanamycin (30 μg), streptomycin (10 μg), and vancomycin (30 μg) were also used as positive controls.

The antibacterial activity of nanofiber membranes was also analyzed quantitatively by the OD technique as described by Akturk (2023).^22^ Tubes without nanofiber samples were used as the control. After incubation, the absorbance of the nutrient broth solutions was measured at 600 nm by using a UV–vis spectrophotometer (BioTek Synergy HT). Bacterial inhibition was calculated as a percentage by using eq 1.
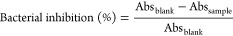
1

The term “Abs_blank_” in the equation is the absorbance value of the control, and the term “Abs_sample_” is the absorbance value of the tubes containing nanofiber samples, after 24 h of incubation at 37 °C. The significant differences among the values were determined using Tukey’s test with the significance level of *p* = 0.05 with SPSS version 27.0.

### 2.6 Cell Cytocompatibility

The fabricated composite nanofiber’s cytotoxicity was evaluated using an MTT assay on L929 cell lines. Briefly, cells were exposed to the tested nanofibers for 24 h, and then they were treated with MTT solution and incubated until formazan crystals formed, which could then be examined. To test the biocompatibility of the Gt–TA–HPO–VAP nanofiber mat, 10^5^ cells were placed in each well of a 96-well plate. The plate was kept in a 5% CO_2_ incubator for 24 h. After that, sterilized Gt–TA–HPO–VAP nanofiber mats were put inside the wells, and after 24 h, MTT solution was added. The absorbance was measured at 570 nm in comparison to a blank (media only). A 1% phenol solution and RPMI were used as positive and negative controls, respectively. The positive control group in MTT analysis is chosen as a substance that is recognized for killing cells. The literature indicates that phenol is toxic to cells. The negative control group should be a component that has no harmful impact on the cells, and the medium in which the cells cultured is the suitable substance. The experiments were performed in three repetitions.

### 2.7 In Vitro Release Tests

The in vitro release study of nanofibers was as per Fahami and Fathi^13^ with modifications. 3 mg of Gt–TA–HPO–VAP nanofibers was placed in a dialysis bag (cellulose membrane, cutoff 12,000 Da, Merck, Germany) with 2 mL of PBS and Tween 80. The bag was placed in a falcon tube containing 20 mL of the same medium, and the tube was gently shaken at 37 °C. Afterward, 1 mL of the medium was taken for analysis at various time intervals (1–6 and 24 h) and refilled with the same quantity to maintain the parameters. The total cumulative quantity of TA (mg), HPO (mg), and VAP (mg) released in the medium volume (mL) was calculated using a UV–vis spectrophotometer (BioTek SynergyHT) at wavelengths of 276, 250, and 330 nm, respectively. The TA, HPO, and VAP release profiles were plotted using zero-order (cumulative percentage of drug released vs time), first-order (log cumulative percentage of drug remaining vs time), and Higuchi model (cumulative percentage of drug released vs square root of time) equations. Linear regression was used to identify the best-fit model.

## 3 Results and Discussion

### 3.1 Morphological Properties

**Figure 2:**
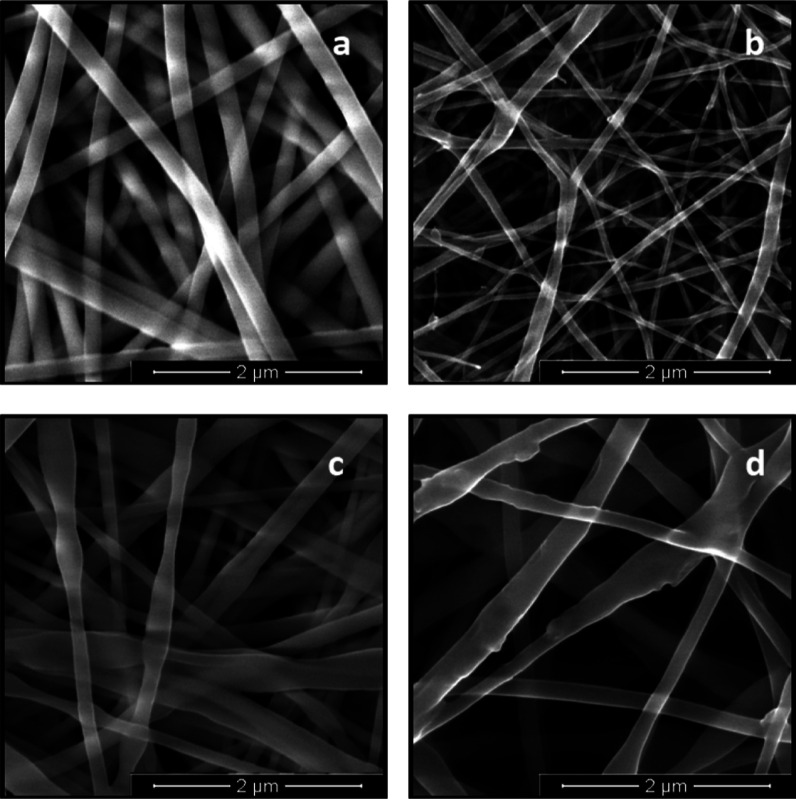
SEM images of Gt (a), Gt–TA (b), Gt–TA–HPO (c), and Gt–TA–HPO–VAP (d) nanofibers.

The morphologies of Gt, Gt–TA, Gt–TA–HPO, and Gt–TA–HPO–VAP nanocomposite fibers are shown in SEM images given in Figure 2. All fibers had a relatively uniform size with no phase separation, indicating that HPO and VAP were successfully incorporated within the fibers. The mean diameters of Gt, Gt–TA, Gt–TA–HPO, and Gt–TA–HPO–VAP nanofibers were 208 ± 34, 73 ± 24, 207 ± 97, and 281 ± 117 nm, respectively. The increase in the nanofibers’ diameter is associated with a change in the electrospinning solution’s conductivity and viscosity.^8^ With the addition of TA in the Gt nanofibers, their size was significantly decreased (*p* < 0.05). This can be attributed to the increase in the electrical conductivity of the Gt solution with the TA integration.^23^ As compared to this, incorporation of HPO and VAP to the solution resulted in a rise in the mean fiber diameter (*p* < 0.05). This might be related to the action of HPO and VAP on reducing the electrical conductivity of Gt solutions, which increased the size of the fibers. Lower electrical conductivity reduces the elongation of the polymer jet by the applied voltage, and thus the fiber diameter increases. These findings were consistent with previous studies on essential oil encapsulation^8,12,18,24,25^ and vitamin encapsulation.^4,11,13,26^ The 3D nanofiber network of Gt–TA–HPO–VAP nanofiber mats was obtained in the range of 128–601 nm. It can be seen that the obtained nanofibers are in the range that is suitable for fibroblast adhesion and proliferation when compared to the three-dimensional network of collagen fibrils and the nanoscale (50–500 nm) range of natural ECM.^18^

### 3.2 Chemical Structure of the Membranes

**Figure 3:**
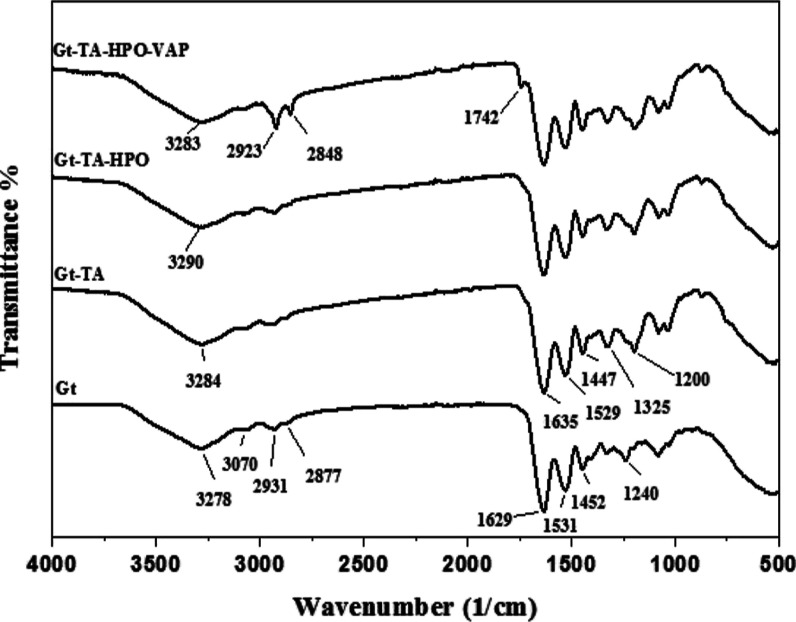
FTIR spectra of Gt, Gt–TA, Gt–TA–HPO, and Gt–TA–HPO–VAP nanofibers.

Figure 3 shows the FTIR analysis findings for Gt, Gt–TA, Gt–TA–HPO, and Gt–TA–HPO–VAP membranes. The amide A peak (NH stretching vibration) at 3278 cm^–1^, the amide I peak (C=O stretching) at 1629 cm^–1^, the amide II peak (NH bending and CH stretching) at 1531 cm^–1^, and the amide III peak (CN stretch and NH in-phase bending) at 1238 cm^–1^ are the characteristic peaks of un-crosslinked Gt nanofibers. Additionally, the band associated with the amide B asymmetric stretching vibration can be seen at 3070 cm^–1^. Stretching vibrations of glycine and proline’s methylene groups were found at 2931 and 2877 cm^–1^, respectively.^27,28^

**Figure 4:**
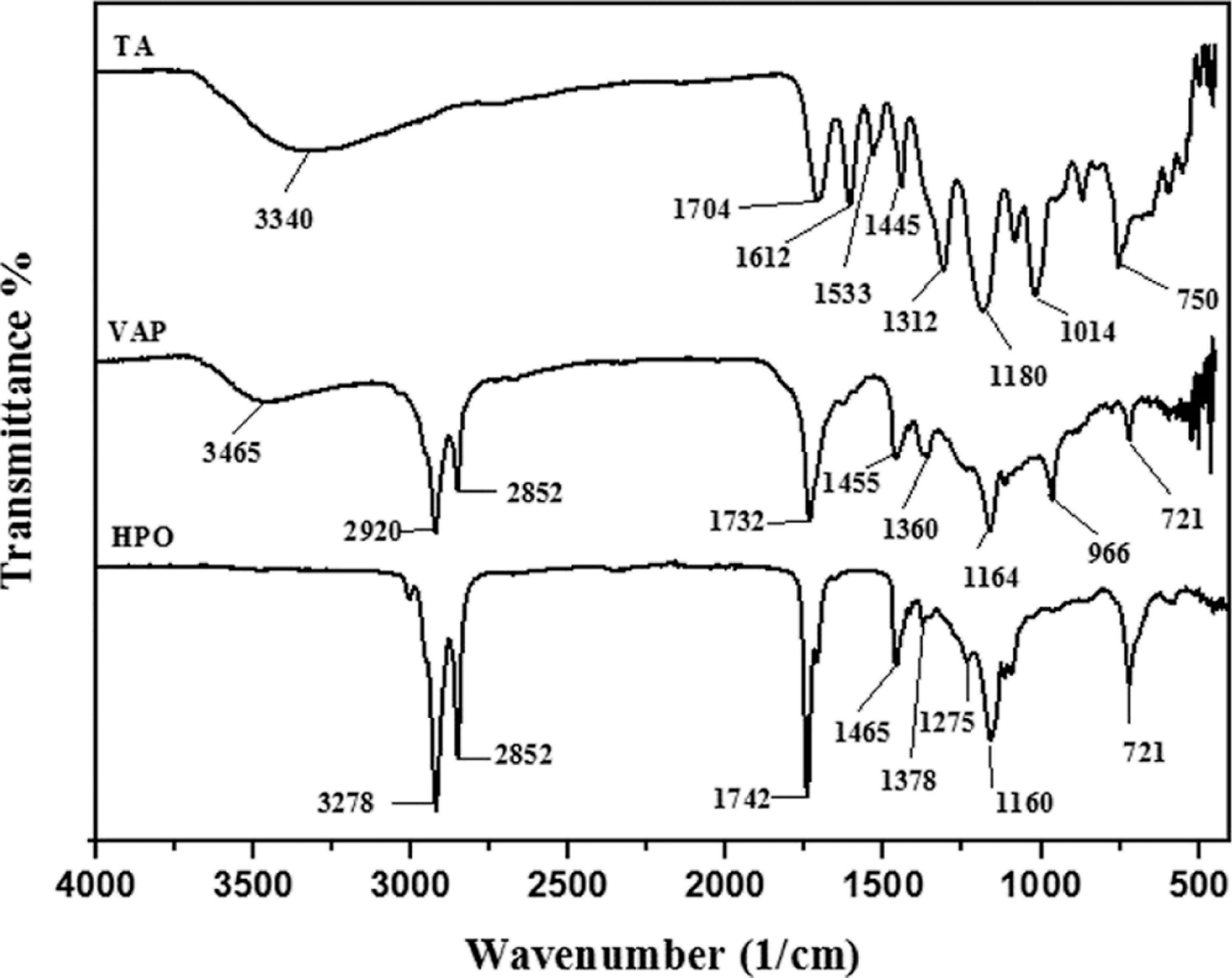
FTIR spectra of TA, HPO, and VAP.

The FTIR spectrum of TA reveals a broader band in the region of 3600–3000 cm^–1^ due to −OH stretching (Figure 4). Bands at 1704 cm^–1^ in the TA spectrum show the presence of the carboxyl carbonyl group. The bands at 1612 and 1533 cm^–1^ indicate the existence of the aromatic ring −C=C–. While the bands at 1445 cm^–1^ correspond to the −C–C– deformation in the phenolic group, and the 1312 cm^–1^ band in the TA spectrum corresponds to the phenolic group. The band at 1180 cm^–1^ is the characteristic band of C–H; moreover, the vibrational bands between 1100 and 1000 cm^–1^ are characteristic bands of C–O and C–H deformation,^29^ respectively.

In the spectra of Gt–TA nanofibers, peaks similar to the spectra of Gt nanofibers were observed, although there were some changes in peaks. The bands at 3278, 1629, 1531, and 1238 cm^–1^ shifted toward 3284, 1635, 1529, and 1200 cm^–1^, respectively, indicating differences in hydrogen bonding and the protein structure. The explanation for the shift of the amide II (NH) band from 1531 to 1529 cm^–1^ in the presence of TA may be the creation of H-bonds between protein and TA molecules, namely, NH···OH and NH···OC (ester) contacts rather than protein–protein and TA–TA interactions.^29,30^ TA is characterized by the presence of 25 hydroxyl and 10 carbonyl groups, while gelatin is known to possess hydrogen bond donors and acceptors. As a result, it is probable that the gelatin chain is cross-linked by TA molecules through the non-covalent interactions of hydrogen bonds.^7,31,32^

HPO contains characteristic C=H methyl stretching bands with peaks at 2852 and 2921 cm^–1^. The carbonyl group (C=O) band with a peak at 1742 cm^–1^ is the ester bond specific to HPO.^19,20,33^ The addition of HPO to Gt–TA membranes resulted in a new peak formation at 1725 cm^–1^ that is assigned to the carbonyl group of HPO in Gt–TA–HPO nanofibers.

The VAP spectra exhibited peaks at 3050 and 966 cm^–1^ from the stretching and bending vibrations of the carbon with a double bond (=CH); 2852 and 2920 cm^–1^ from the stretching for free CH_3_ groups; 1732 cm^–1^ from the carbonyl group (C=O) stretching for ester; and different peaks from 1164 to 1455 cm^–1^ that are typical of the CO group.^14,34,35^ Moreover, new peaks appeared at 2848, 2923, and 1742 cm^–1^ wavelengths with the addition of VAP to Gt–TA–HPO nanofibers. This confirmed the presence of both CH and carbonyl groups in Gt–TA–HPO–VAP nanofibers. These findings indicated that HPO and VAP were loaded into the membranes and that components incorporated into the membrane did not alter their chemical structure as a result of solvent interactions.

### 3.3 Thermogravimetric Analysis

**Figure 5:**
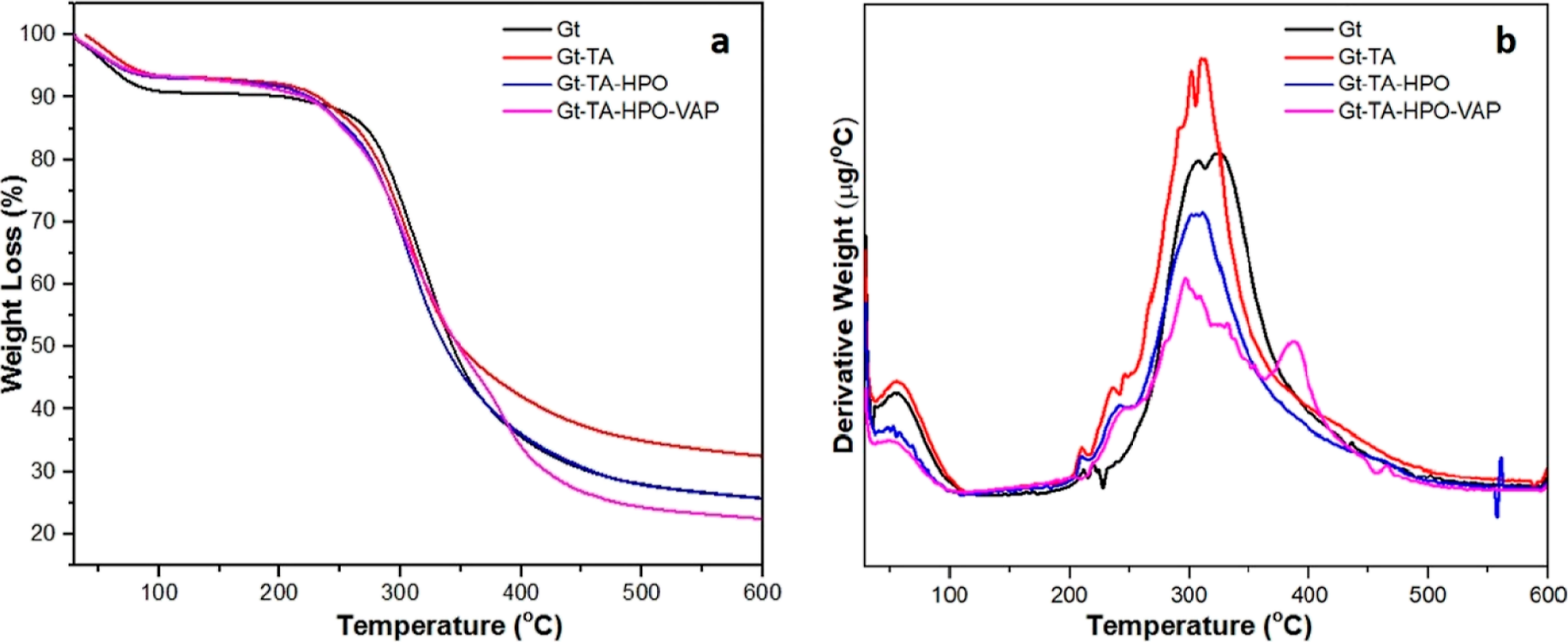
TGA (a) and DTG (b) analysis of Gt, Gt–TA, Gt–TA–HPO, and Gt–TA–HPO–VAP nanofibers.

The thermal stabilities of Gt, Gt–TA, Gt–TA–HPO, and Gt–TA–HPO–VAP nanofiber membranes were investigated using TGA (Figure 5a) and DTG (Figure 5b). The degradation temperature ranges of the nanofiber membranes are given in Table 1. The DTG of Gt, Gt–TA, and Gt–TA–HPO nanofiber membranes showed two-step degradation trends. The first decomposition range (*T*_d1_) indicated the elimination of physically adsorbed water molecules. The second decomposition range (*T*_d2_) was related to the breakdown of proteins and the rupturing of low-energy intermolecular interactions.^36^ The loss of the glycerol molecule from high-molecular-weight protein fractions is another reason for the change in this region.^7^ Weight loss of Gt nanofibers in the *T*_d1_ and *T*_d2_ regions was obtained as 10 and 65%, respectively. When TA was utilized as the cross-linking agent, weight loss of Gt–TA nanofibers in the *T*_d1_ and *T*_d2_ regions was reduced to 8 and 32%, respectively. The hydroxyl and ester groups of TA are considered to break down in the *T*_d2_ range. These findings indicated that Gt–TA nanofibers have greater thermal resistance because of TA function as a cross-linker.^7,37^ In addition, Gt–TA–HPO nanofibers degraded similar to Gt–TA nanofibers for the *T*_d1_ range, but a weight loss of around 65% was observed in the *T*_d2_ range. The Gt–TA–HPO–VAP nanofiber mat showed three decomposition steps. The third decomposition range *T*_d3_ indicated the contribution of VAP. The weight loss of the membrane at the *T*_d1_, *T*_d2_, and *T*_d3_ ranges was about 7, 48, and 22%, respectively. It has been demonstrated in the literature that the thermal breakdown of the chains of VAP causes the mass loss at this temperature range.^38^ The DTG results made it more obvious how the addition of VAP reduced the rate of thermal breakdown and promoted thermal stability.

**Table 1:** Degradation Temperatures of Gt, Gt–TA, Gt–TA–HPO, and Gt–TA–HPO–VAP Nanofiber Membranes^a^

	*T*_d1_ (°C)			*T*_d2_ (°C)			*T*_d3_ (°C)		
sample	*T*_onset_	*T*_max_	*T*_end_	*T*_onset_	*T*_max_	*T*_end_	*T*_onset_	*T*_max_	*T*_end_
Gt	36.9	53.2	123.4	178.8	329.2	557.7			
Gt–TA	35.8	53.7	113.4	171.1	321.9	596.5			
Gt–TA–HPO	43.1	53.3	111.7	184.1	311.9	563.8			
Gt–TA–HPO–VAP	42.1	56.7	108.2	212.6	295.7	366.3	366.3	390.6	537.6

a *T*_d1_: first decomposition range, *T*_d2_: second decomposition range, *T*_d3_: third decomposition range.

### 3.4 Antibacterial Activity

**Figure 6:**
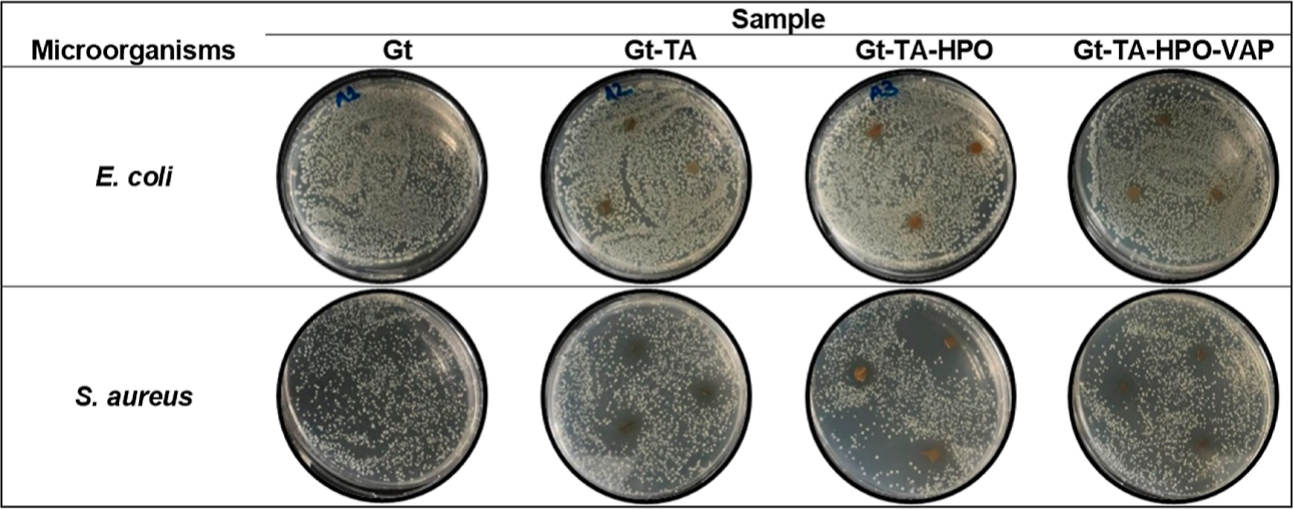
Disc diffusion assay of Gt, Gt–TA, Gt–TA–HPO, and Gt–TA–HPO–VAP nanofibers.

Figure 6 and Table 2 show the antibacterial test results of the Gt, Gt–TA, Gt–TA–HPO, and Gt–TA–HPO–VAP nanofibers with disc diffusion assay against *E. coli* and *S. aureus*. It has been shown that Gt nanofibers were dissolved completely and did not have any antibacterial activity, which was also known from the literature.^7^ The addition of TA, which was used as a cross-linking agent, into Gt nanofibers provided antibacterial activity against *E. coli* and *S. aureus*, consistent with the literature.^7,39^

**Table 2:** Antibacterial Test Results for the Nanofibers and Positive Controls on *E. coli* and *S. aureus*^a^

	inhibition zone diameters including disc diameter (mm)	
sample	*E. coli*	*S. aureus*
Gt	0^a^	0^a^
Gt–TA	6.50 ± 0.5^b^	14.50 ± 2.21^b^
Gt–TA–HPO	7.83 ± 1.34^b^	17.00 ± 0.63^b^
Gt–TA–HPO–VAP	6.67 ± 0.94^b^	15.33 ± 2.42^b^

	inhibition zone diameters including disc diameter (mm)	
standard antibiotic discs	*E. coli*	*S. aureus*
ampicillin	19.0 ± 0.0	17.5 ± 0.5
gentamycin	24.0 ± 2.0	9.0 ± 1.0
kanamycin	25.5 ± 0.5	14.5 ± 3.5
streptomycin	22.5 ± 0.5	22.0 ± 0.0
vancomycin	6.0 ± 0.0	22.0 ± 0.0

a,b Inhibition zone diameters sharing the same letter are not significantly different at *p* > 0.05, *n* = 3.

TA causes the death of bacteria by attacking the bacterial cell membranes through hydrophobic–hydrophobic interactions, which result in the breakdown of the membrane and the release of essential components from the bacterial cells.^23^ The inhibition zones around the Gt–TA, Gt–TA–HPO, and Gt–TA–HPO–VAP nanofibers were not significantly different against *E. coli* and *S. aureus* (*p* < 0.5).

**Figure 7:**
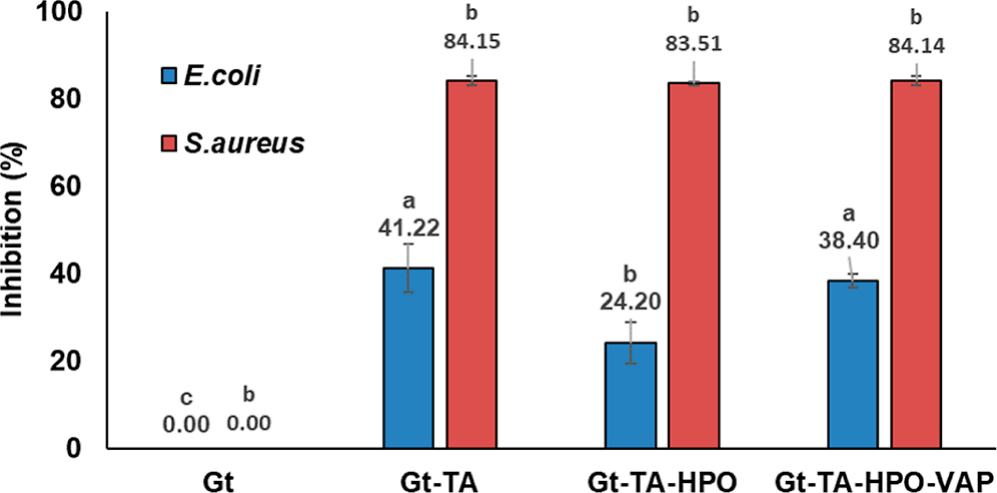
Antibacterial activity of the nanofibers *Bars sharing the same letter are not significantly different for each microorganisms at *p* > 0.05, *n* = 3.

Quantitative analysis results of the nanofiber samples can be seen in Figure 7. It can be demonstrated that nanofibers were more effective against *S. aureus* than *E. coli* in all nanofibers. That might be due to the gram (−) bacteria peptidoglycan layer.^40,41^ Additionally, Gt–TA–HPO nanofibers have significantly lower antibacterial activity against *E. coli*.

The comparison of the effectiveness of the membranes against *E. coli* reveals that HPO addition reduces the antibacterial activity. Orhan et al. investigated in vitro antimicrobial and antiprotozoal activity properties with various St. John’s Wort oils. They found that *H. perforatum* macerate oils did not demonstrate substantial antibacterial action, similar to our study.^42^ It was also observed that VAP addition had an increasing antibacterial effect against *E. coli* on the Gt–TA–HPO–VAP nanofibers.^4^ Moreover, the antibacterial activity of the membranes was compared with the antibacterial activity of various antibiotic discs (disc diffusion plates for antibacterial activity of antibiotic tests are given in the Supporting Information). The results show that the obtained membranes have an efficiency against *S. aureus* that is comparable to that of some antibiotics (gentamycin, kanamycin). Thus, it can be suggested that Gt–TA, Gt–TA–HPO, and Gt–TA–HPO–VAP nanofibers indicated enough antibacterial behavior as potential wound dressing materials. Therefore, the Gt–TA–HPO–VAP nanofiber mat was chosen for in vitro release and cytotoxicity tests since this mat contains the natural therapeutic wound healing agents in addition to its antibacterial effect.

### 3.5 In Vitro Release Study

**Figure 8:**
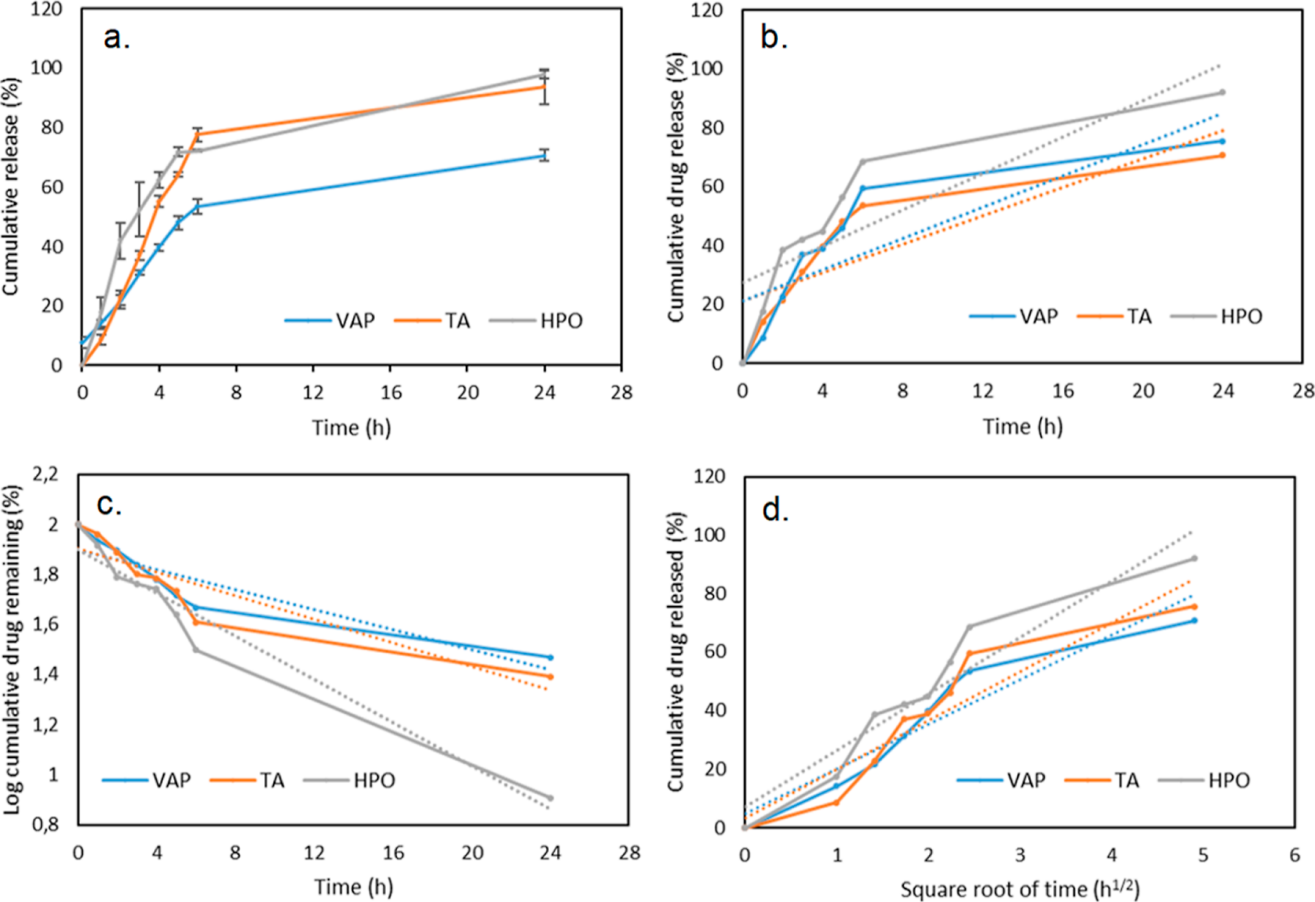
Release patterns of VAP, TA, and HPO from Gt–TA–HPO–VAP nanofibers: (a) cumulative release, (b) zero-order model, (c) first-order model, and (d) Higuchi model.

The in vitro release profiles of TA, HPO, and VAP of the Gt–TA–HPO–VAP nanofiber mats are shown in Figure 8. The Gt–TA–HPO nanofiber mat released 53, 59, and 68% of the VAP, TA, and HPO, respectively, over the first 6 h. Then, the released VAP, TA, and HPO increased gradually in a sustained manner up to 24 h. The results indicated that 70, 75, and 92% of VAP, TA, and HPO, respectively, were released from the nanofiber mat within 24 h.

Drug release behaviors of VAP, TA, and HPO in 24 h were evaluated by various drug delivery release mechanisms. According to release kinetics, the data were collected and are given in Table 3. The data revealed that the zero-order was not suitable for the release data. The data of VAP and TA release profiles were fitted to the Higuchi model, indicating that VAP and TA releases were based on a diffusion process based on Fick’s law. In contrast, the HPO release profile fitted to the first-order kinetic model, demonstrating that HPO release from the composite was concentration-dependent.^43^ Additionally, the release of TA from the nanofiber mat indicated that partial crosslinking occurred between Gt and TA due to weak non-covalent interactions of hydrogen bonds.

**Table 3:** Kinetic Data of the In Vitro Release of VAP, TA, and HPO from the Gt–TA–HPO–VAP Nanofibers

	zero-order		first-order		Higuchi	
	*K*	*r*^2^	*K*	*r*^2^	*K*	*r*^2^
VAP	2.4117	0.6580	0.0200	0.8141	15.242	**0.8934**
TA	2.6478	0.6550	0.0235	0.8266	16.632	**0.8785**
HPO	3.0786	0.6871	0.0431	**0.9518**	19.725	0.9155

### 3.6 Cytotoxicity

**Figure 9:**
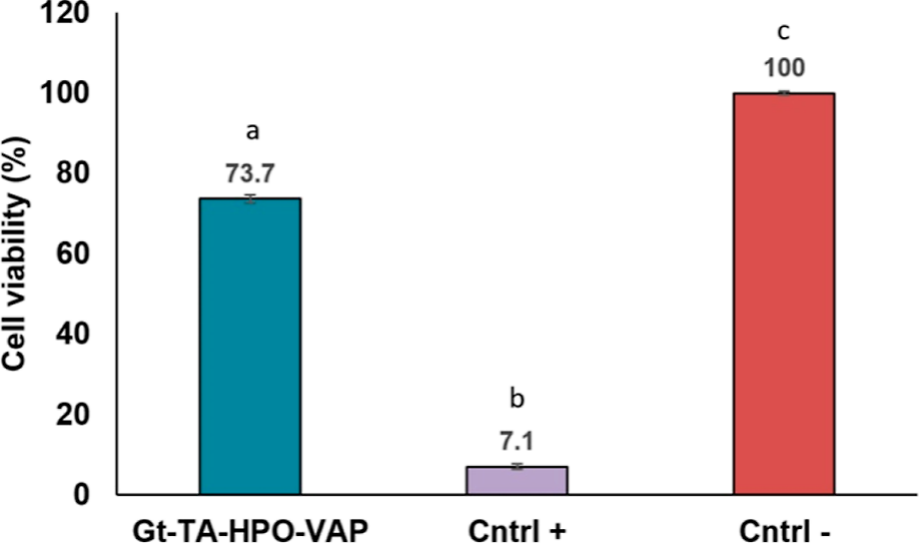
Cell viability of Gt–TA–HPO–VAP nanofibers (1% phenol solution and RPMI were used as positive (cntrl +) and negative control (cntrl −), respectively). Bars sharing the same letter are not significantly different at *p* > 0.05, *n* = 3.

The cytotoxic effects of materials on healthy cells are another critical feature of materials in the biomedical area. Here, the viability of the L929 cell line after 24 h of incubation was used to measure the cytotoxicity of the Gt–TA–VAP–HPO nanofibers (Figure 9). According to ISO-10993-5, cell viability higher than 80% is considered as non-toxic, between 80 and 60% is considered weakly toxic, between 60 and 40% is considered moderately toxic, and less than 40% is considered highly toxic.^44^ With 73.7% cell viability, Gt–TA–VAP–HPO nanofibers showed a moderate cell proliferation rate. Eğri and Erdemir electrosprayed St. John’s Wort-doped PEG onto a polycaprolactone (PCL) nanofiber membrane using electrospraying in their study. As a result of in vitro tests, they determined that this material was biocompatible for L929 fibroblast cells.^19^ Afterward, they performed in vivo tests of this membrane with Wistar Albino rats. They found that this membrane shortened the healing time, and significant healing was observed especially in membranes containing *H. perforatum* oil during the therapy process.^5^ Similarly, Guleken et al. conducted in vivo tests with Wistar albino male rats using aloe vera and *H. perforatum* oil-doped poly (ϵ-caprolactone)/gelatin nanofibers and observed that HPO loading was a better alternative for diabetic wounds than aloe vera.^45^ However, VAP has a dose-dependent wound healing activity.^46^ Although, the cell proliferation of the Gt–TA–HPO–VAP nanofiber mat is in an acceptable range, it can be said that an optimization study might be carried out to increase the cell proliferation of the nanofiber mats by changing the VAP amount. Considering the improved healing effect of HPO in diabetic wounds,^33,45,47^ the Gt–TA–VAP-HPO nanofiber mat with its moderate cell proliferation rate might be employed as a biocompatible wound dressing material.

## 4 Conclusions

This study reports the preparation of Gt nanofibers incorporated with TA, HPO, and VAP with a proper fiber size for tissue engineering applications through the electrospinning technique. To the best of our knowledge, this is the first report on the participation of TA, HPO, and VAP with Gt in electrospun nanofibers for wound healing purposes. Based on the quantity of Gt, 5 and 50% of VAP and HPO, respectively, were loaded in the Gt solution (15% w/v in 4:6 v/v acetic acid/deionized water). The detailed characterization of the obtained samples including SEM, FTIR, TGA, in vitro release, antibacterial, and cell cytocompatibility studies were performed. These findings indicated that using a natural crosslinking agent (TA) in combination with environmentally friendly solvents (acetic acid and deionized water) and natural therapeutic wound healing agents (VAP and HPO) in Gt nanofibers improved the properties of Gt nanofiber, resulting in an environmentally compatible, cost-effective nanofiber structure as a candidate for wound healing applications. Moreover, based on these results, additional optimization and characterization studies (wound healing, tissue or organ compatibility tests) might be conducted to develop suitable wound dressings for future studies.

## Supporting Information Available

Disc diffusion assay plates for antibacterial activity of antibiotic discs (ampicillin, gentamycin, kanamycin, streptomycin, and vancomycin) against *E. coli* and *S. aureus* (PDF)
