# Supplementary material for: Hypericum perforatum Oil and Vitamin A Palmitate-Loaded Gelatin Nanofibers Cross-Linked by Tannic Acid as Wound Dressings
Source: ACS Omega. 2023 Jun 26;8(26):24023–31. doi: 10.1021/acsomega.3c02967 (PMC10324379; doi:10.1021/acsomega.3c02967)
Supplement: Supplementary file 1 — ao3c02967_si_001.pdf [file ao3c02967_si_001.pdf]

Supporting Information for Review Only

Hypericum perforatum Oil and Vitamin A Palmitate Loaded Gelatin Nanofibers Cross-linked by Tannic Acid as Wound Dressings

Aysen Akturk[1*], Funda Nur Kasikci[1], Dilara Nur Dikmetas[2], Funda Karbancioglu-Guler[2], and Melek Erol-Taygun[1]

[1]Department of Chemical Engineering, Istanbul Technical University, Istanbul, Turkey

[2]Department of Food Engineering, Istanbul Technical University, Istanbul Turkey

*Corresponding Author, e-mail: akturkay@itu.edu.tr

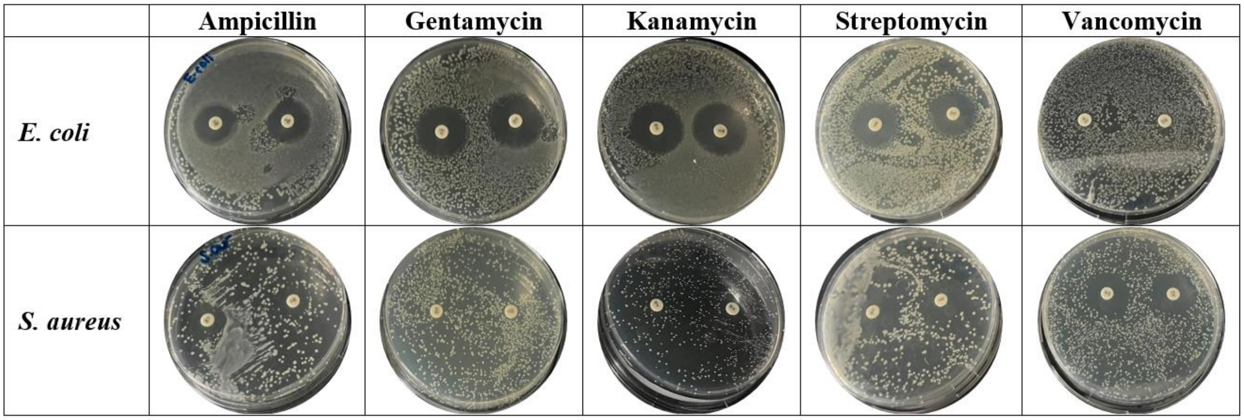

Figure S1. Antibacterial activity of antibiotic discs against *E.coli* and *S. aureus*
